# Smoking Cessation after a Cancer Diagnosis: A Cross-Sectional Analysis in the Setting of a Developing Country

**DOI:** 10.3390/clinpract11030067

**Published:** 2021-08-10

**Authors:** Marin Golčić, Ilijan Tomaš, Aleksandra Stevanović, Goran Golčić, Renata Dobrila-Dintinjana, Suzana Erić, Mirela Šambić-Penc, Martina Baretić Marinac, Lidija Gović-Golčić, Tea Majnarić

**Affiliations:** 1 Clinical Hospital Center Rijeka, Department of Radiotherapy and Oncology, 51000 Rijeka, Croatia; goran.golcic@gmail.com (G.G.); renatadobrila@windowslive.com (R.D.-D.); 2 Clinical Hospital Center Osijek, Department of Radiotherapy and Oncology, 31000 Osijek, Croatia; tomas.ilijan@kbo.hr (I.T.); eric.suzana@kbo.hr (S.E.); mirela.penc@gmail.com (M.Š.-P.); 3 School of Medicine, University of Osijek Josip Juraj Strossmayer, 31000 Osijek, Croatia; 4 Department of Basic Medical Sciences, Faculty of Health Studies, University of Rijeka, 51000 Rijeka, Croatia; stevanovic.aleksandra@gmail.com; 5 Department of Psychiatry and Psychological Medicine, School of Medicine, University of Rijeka, 51000 Rijeka, Croatia; 6 Family Medicine Practice, Community Health Center of Primorsko-Goranska County, 51000 Rijeka, Croatia; martinab13@gmail.com (M.B.M.); lidijagg@gmail.com (L.G.-G.); 7 Institute of Emergency Medicine of Primorsko-Goranska County, 51000 Rijeka, Croatia; tea.majnaric19@gmail.com

**Keywords:** breast cancer, cancer, public health, smoking, smoking cessation

## Abstract

Since smoking accounts for around 30% of all cancer deaths, public health campaigns often focus on smoking cessation as a means of primary prevention. However, smoking after cancer diagnosis is also associated with a higher symptom burden and lower survival rate. As data regarding smoking cessation vary dramatically between different populations, we aimed to analyze smoking prevalence in cancer patients, smoking cessation after cancer diagnosis, and the factors associated with smoking cessation in the setting of a developing country. We performed a cross-sectional survey on 695 patients in two clinical hospital centers. After cancer diagnosis, 15.6% of cancer patients stopped smoking. Male gender, younger age, and smoking-related cancer were the main factors associated with greater smoking cessation (*p* < 0.05). A total of 96% of breast cancer patients continued to smoke after cancer diagnosis and, compared to lung and colorectal cancer patients, exhibited a lower reduction in the number of cigarettes smoked (*p* = 0.023). An alarming rate of smoking prevalence was recorded in younger patients (45.6% at the time of cancer diagnosis) suggesting a future rise in smoking-related cancers and complications. These results should guide anti-smoking public health campaigns in transitional countries with a critical focus on younger and breast cancer patients.

## 1. Introduction

Smoking is thought to cause around 30% of all deaths from cancer, including 87% of all deaths due to lung cancer [1]. Although public health campaigns often focus on smoking cessation as a means of primary prevention, not enough emphasis is placed on quitting smoking after the cancer diagnosis. Cigarette smoking during cancer treatment is associated with more severe symptoms, a higher chance of developing another primary tumor, and a decreased survival rate, whereas patients who quit smoking after a cancer diagnosis exhibit a longer survival rate [2,3,4,5].

A large percentage of patients diagnosed with cancer do not quit smoking during cancer treatment, partially due to the low perceived danger of smoking [6]. Tseng et al. reported that just less than two-thirds of cancers survivors continued to smoke after being diagnosed with cancer, similar to Hewitt et al., who showed that 19.7% of cancer survivors actively smoke [7,8]. On the contrary, Hawkins et al. discovered that almost 80% of cancer survivors never smoked [9], showing heterogeneity in smoking prevalence and cessation between different populations.

Tseng et al. also showed that smoking continuation is associated with a younger age, female gender, and having a non-smoking related cancer [7]. However, it is important to look at this data through the prism of absolute numbers, as non-smoking related cancers such as breast and prostate cancer patients also had the lowest smoking prevalence (8–14% [10,11,12] and 5.5–8% [10,13], respectively). On the other hand, patients with gynaecological cancers (37–46%) and other typically smoking-related malignancies, including lung, larynx, and pharynx cancers, reported a higher prevalence of smoking (around 20%) [11,13,14]. Furthermore, being younger than 40 at the time of cancer diagnosis is associated with a 70% higher chance of actively smoking than similarly aged patients with no cancer [15]. Other studies also confirmed higher smoking prevalence in the youngest cancer survivor group (37–43%) [11,13].

Currently, we have no data for smoking prevalence or smoking cessation following the diagnosis of cancer in Croatia or other transitional countries. The Croatian Institute of Public Health surveyed tobacco use in the general population in 2015, reporting that 35% of men and 27% of women were active smokers, consuming around 15 to 24 cigarettes a day on average [16]. According to the Global Adult Tobacco Survey, the percentage of female smokers is among the highest globally reported [17].

Our aim was to evaluate smoking prevalence in cancer patients and the effect of smoking cessation after a cancer diagnosis in the Croatian population. We hypothesized that, in the Croatian population, a cancer diagnosis is associated with smoking cessation.

## 2. Materials and Methods

The research had a cross-sectional design and was undertaken in the outpatient clinic of the oncological department in the Clinical Hospital Center Rijeka from 2016 to 2018 and later expanded to combine data from Clinical Hospital Center Osijek. Hospitals are located in cities with over 100,000 residents and are the primary cancer centers for approximately 500,000 people. Inclusion criteria were: over 18 years of age, ability to read and understand written questions, undergoing active oncological treatment, and willingness to participate in the research.

A simple questionnaire was constructed in order to increase compliance; patients were required to answer several demographic questions and a total of 7 smoking-related questions (Appendix A). Only patients who answered the question: “Have you ever been a smoker?” were taken in for further analysis. The questionnaire was intened not to be anonymous in order to acquire accurate data regarding the cancer stage and type for further analysis, although several patients insisted on anonymity. All the patients with the appropriate inclusion criteria in our outpatient clinic were offered the questionnaire, although the exact number of refusals was not noted. The time period of the questionnaire administration was prolonged to due technical issues (sick leave and change of staff).

The questionnaire was administered after screening for the inclusion criteria by a trained nurse and study doctors and was filled out in less than 5 minutes. No issues were reported with the questionnaire, although 69 patients left out the information regarding the highest level of education, 40 patients did not answer the question regarding the frequency of previous smoking, and individual patients left out the information regarding age and gender. 

Exact McNemar’s tests were run to determine if there was a difference in the proportion of non-smokers before and at the time of diagnosis and between the time of diagnosis and during treatment. Chi-square tests were used to determine the differences in smoking status and the number of cigarettes at a specific time. Standard descriptive statistics were used to describe the population. All statistics were performed in Statistica 10 (StatSoft, Inc., Tulsa, OK, USA).

The research design and methods were modified per suggestions by the Ethical Committee of the Clinical Hospital Center Rijeka, and the research was approved on 29 April 2016 (class: 003-05/16-1/20), while the retrospective use of the data regarding smoking habits was confirmed by the Ethical Committee of Clinical Hospital Center Osijek 12 September 2019 (class: R2-12487/2019).

## 3. Results

### 3.1. Demographic Data

A total of 695 cancer patients were screened using the questionnaire. The average age was 61.1 ± 11.2 years (median: 62, range 18–89), while the most common primary cancer sites were breast (*N* = 168), lung (*N* = 127) and colorectal cancer (*N* = 103). Demographic data are summarized in Table 1.

### 3.2. Smoking Cessation and Associated Factors

Only participants who declared to have smoked at any point in their life (*N* = 441, 63% of the total) were included in further analysis. At the time of the cancer diagnosis, 211 patients (47.8% of all the smokers) had already quit smoking and 230 (52.2%) were still smoking. After the cancer diagnosis, 48 of the active smokers (20.9%) stopped smoking, and 158 (68.7%) reduced the number of cigarettes smoked, while 23 did not change their smoking pattern, and one patient did not answer the question. However, 12 patients who did not smoke at the time of the cancer diagnosis reported smoking during the cancer treatment (5.2%). Hence, there was a net change in smoking cessation in 15.6% of smokers after the cancer diagnosis.

**Figure 1:**
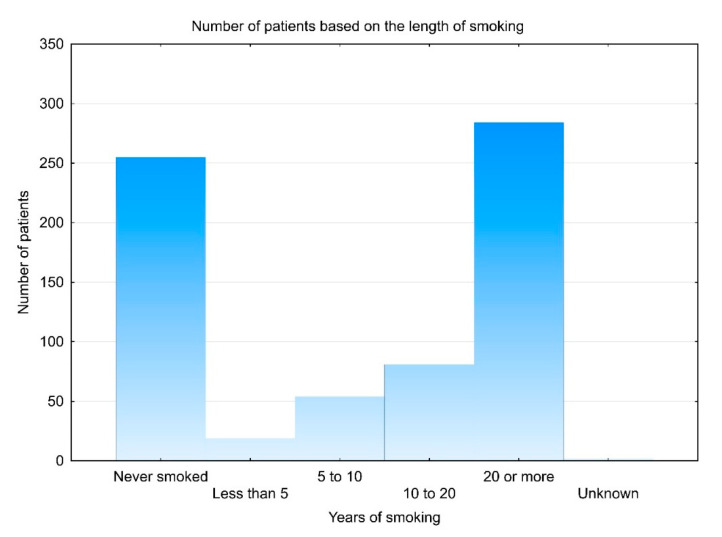
Number of patients based on the length of smoking in years. A similar number of patients reported never smoking (*N* = 255) and smoking 20 years or more (*N* = 284); both much higher numbers compared to patients who smoked for less than 5 years (*N* = 19), from 5 to 10 years (*N* = 52) and from 10 to 20 years (*N* = 81). No data is available for four patients.

Regarding the length of smoking, 19 patients (2.7%) smoked for less than 5 years, 54 (7.8%) smoked for 5 to 10 years, 81 (11.6%) smoked from 10 to 20 years, while the majority of patients (*N* = 285, 41.0%) smoked for 20 or more years (Figure 1). Two patients did not disclose such information.

We found no significant differences in smoking status regarding the level of education, possibly due to the small sample size, as there was a trend showing college-educated patients had the lowest percentage of ever-smokers. However, a higher proportion of high-school educated patients quit smoking after the cancer diagnosis compared to patients with other levels of education (*p* < 0.001) (Table 2).

The percentage of ever-smokers was higher among men than women (75% vs. 53% of the total number of patients, Table 1). However, a similar percentage in both genders still smoked at the time of a cancer diagnosis (Table 2), indicating that a higher proportion of males quit smoking following a cancer diagnosis (*p* < 0.001). In total, 94% of female smokers continued to smoke after a cancer diagnosis, opposed to 77% of male smokers.

A total of 72% of patients younger than the median age of 62 were smoking at any period, compared to 56% of older patients. Although a higher frequency of younger patients quit smoking after a cancer diagnosis (*p* = 0.001), more than half of younger ever-smokers continue to smoke during the cancer treatment (52%) (Table 2).

We further analyzed only the patients with the three most common types of cancer (lung, breast, and colorectal cancers). When observing smoking by cancer type, the lowest rate of smoking prevalence was recorded in ovarian and breast cancer patients (42% and 49%, respectively), while oropharyngeal (100%) and lung cancer patients (92%) reported the highest percentage of previous smoking. Among patients who reported smoking at any period, 56% of lung and 57% of breast cancer patients were also actively smoking at the time of the cancer diagnosis, compared to only 35% of colorectal cancer patients (*p* = 0.015). After the cancer diagnosis, there was a change in the smoking cessation patterns based on cancer type as 77% of lung cancer patients continued to smoke, compared to 96% of breast cancer patients and 90% colorectal cancer patients (*p* = 0.001, Table 2).

Similar results were found for patients with smoking-related cancers who exhibited a higher smoking rate at the time of the cancer diagnosis (60% vs. 48%, *p* = 0.011) than patients with less or non-smoking related cancers. However, after the cancer diagnosis, the proportion of non-smokers among smoking-related cancer patients increased by 14%, compared to less/non-smoking related cancer patients, where it had increased by only 5% (Table 2).

The presence of metastatic cancer was correlated with a greater proportion of patients smoking during the cancer diagnosis (*p* = 0.01), but not during cancer treatment (*p* = 0.551), most likely due to the type of cancer, since only 22% of patients with breast cancer were metastatic when the questionnaire was applied, compared to 65% of lung cancer patients. The proportion of non-smokers increased significantly in the metastatic group (13%), but not in the non-metastatic group (3%) (Table 2).

### 3.3. Number of Cigarettes Smoked before and after Cancer Diagnosis

**Figure 2:**
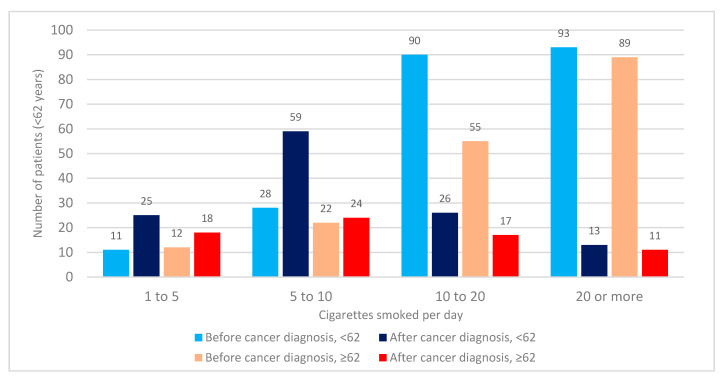
Analysis of the average number of cigarettes smoked per day based on median age at any time before cancer diagnosis and during the cancer treatment. The blue-colored columns indicate patients under the median age of 62, while the red-colored columns include patients older than 62 years. Statistical significance in number of cigarettes smoked is registered only after cancer diagnosis (χ^2^ = 17.085, *p* = 0.004; U = 20287.000, *p* = 0.001).

The majority of our patients reported previously smoking 20 or more cigarettes a day (*N* = 182, 41.3% of ever-smokers), with almost three-quarters of smokers consuming over 10 cigarettes a day (74.1%) (Figure 2). The cancer diagnosis was followed by a significant change in smoking habits, with only 67 patients (15.2%) consuming over 10 cigarettes a day during the cancer treatment. In total, 69% of the patients reduced the number of cigarettes per day, and although 12 patients restarted smoking, none of the smokers admitted to an increase in the total number of cigarettes smoked.

There were no differences in smoking habits between patients younger and older than the median age of 62 (*p* = 0.978). Although both groups reduced the average number of cigarettes smoked during the cancer treatment, the reduction is more significant in younger patients, 32% of whom report smoking more thane 10 cigarettes a day compared to 40% of older patients (*p* = 0.001) (Figure 2).

**Figure 3:**
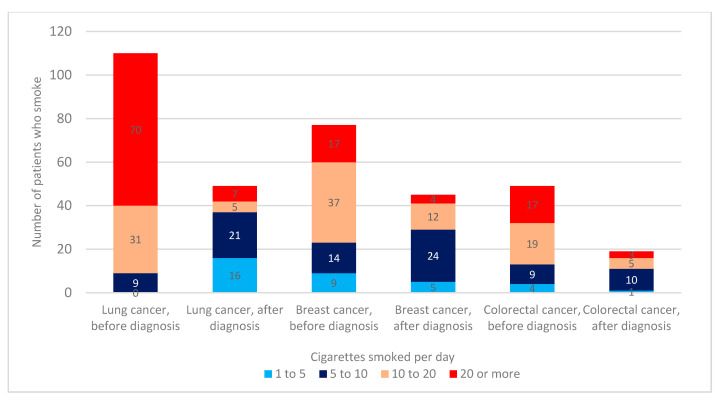
Analysis of the average number of cigarettes smoked per day based on the three most common cancer types at any time before cancer diagnosis and during the cancer treatment. Statistical significance in number of cigarettes smoked is registered both before (χ^2^ = 50.006, *p* = <0.001; H = 36.926, *p* = <0.001) and after cancer diagnosis (χ^2^ = 20.498, *p* = 0.009; H = 7.554, *p* = 0.023).

Prior to the cancer diagnosis, more than 10 cigarettes per day were smoked by 70%, 73%, and 92% of breast, colorectal, and lung cancer smoker-patients, respectively. However, during the cancer treatment, the ratio reversed, with 36% of breast cancer, 42% of colorectal cancer, and 24% lung cancer patients reporting smoking >10 cigarettes a day (Figure 3).

**Figure 4:**
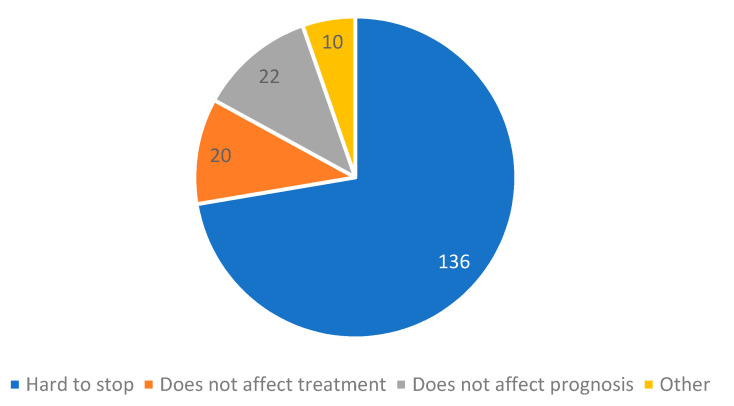
Number of patients based on the given reason for smoking continuation. More than one answer was allowed.

Finally, we asked our patients who still smoked the reason for the continuation of smoking, allowing more than one answer. Only 11% (*N* = 20) stated they did not believe smoking affected treatment and a similar number (*N* = 22, 12%) stated that they believed smoking did not affect prognosis, with 10 patients (5%) stating ‘other’ as a reason for not stopping (Figure 4).

## 4. Discussion

The majority of studies that analyzed smoking cessation and prevalence after a cancer diagnosis were performed on the Western population. However, transitional countries such as Croatia exhibited different trends in smoking patterns, with a significantly higher number of smokers, especially in women [16]. Our data showed that 63.4% of our patients admitted to regular smoking before the cancer diagnosis, with around half of the patients quitting smoking at any period before the cancer diagnosis (47.8%), similar to previous reports [7].

Although over two-thirds of smokers reduced the volume of daily cigarettes, cancer diagnosis caused a net change in smoking cessation in only 15.6% of smokers. This percentage of smokers who continued to smoke during the cancer treatment (84.4%) is around 20% higher than previously reported by Tseng and colleagues for the Western population [7].

We found four main factors associated with a change of proportions in smoking habits: education, gender, age, and cancer type. Although there was a trend of lower smoking prevalence in college-educated patients, only high school education was associated with an increased proportion of smoking cessation, possibly due to disproportionate sample sizes. We also recorded that while smoking is more prevalent in men, a higher percentage of males quit smoking after the cancer diagnosis, similar to previous reports [7].

Our research additionally confirmed the alarming rate of smoking habits in patients younger than the median age of 62, 72% of whom smoked at any time in life. Although a higher proportion of younger patients quit smoking following a cancer diagnosis, a staggering 52% of all younger smokers still smoked during active cancer treatment. If our data accurately represented the Croatian population, we can expect a rise in smoking-related cancers, comorbidities, and complications during treatment in Croatia as that younger population reaches the median age of cancer incidence (62 years in our study).

The type of primary cancer also affects smoking cessation, as a higher proportion of cancer patients with smoking-related malignancies quit cigarettes compared to patients with less-smoking-related cancers (76% vs. 91%, respectively). A high rate of smoking continuation for less-smoking-related cancers was mainly due to breast cancer patients. Although only 49% of breast cancer patients used to smoke at any time point and half of them quit smoking previous to the cancer diagnosis, a surprising 96% of patients continued smoking following the diagnosis with breast cancer, a significantly higher proportion compared to 77% of lung cancer patients (*p* = 0.001).

Low smoking cessation in breast cancer patients is a known issue, as Persson et al. reported that in the Swedish population, only 10% of breast cancer patients stopped smoking in the first year after their cancer surgery [18]. The issue with low smoking cessation is less pronounced in Western countries because only 8 to 14% [10,11,12] of breast cancer patients are active smokers, compared to 28% in our research. Additionally, breast cancer patients had a lower probability of reducing the number of cigarettes smoked per day than lung cancer patients (Figure 4). The value of smoking cessation in breast cancer survivors is being discussed more often as various studies showed that female patients who did not change their smoking habits following a cancer diagnosis had a higher likelihood of dying of breast cancer (HR 1.71–2.01). In contrast, women who gave up smoking after being diagnosed with cancer had a lower chance of dying from breast and respiratory cancer [19,20]. Persson and et al. additionally showed that patients older than 50 who used aromatase inhibitors and continued smoking had a significantly higher risk of events associated with breast cancer (HR 2.98), disease progressing to the metastatic stage (HR 4.19), and dying (HR 3.52), suggesting a possible interaction of smoking and commonly used aromatase inhibitors. Hence, although breast cancer patients were not typically the focus of anti-smoking campaigns, smoking cessation during cancer treatment could potentially result in a longer survival rate, especially in transitional countries with a large percentage of female smokers such as Croatia.

Finally, our research showed that only 11% of patients did not believe smoking cessation affected their prognosis, and another 12% did not believe it affected their treatment (Figure 4), indicating that patients were aware of the dangers of smoking. Such data are in concordance with the Croatian survey on tobacco use [16] and could point to the fact that emphasis should be placed on offering additional treatment options for quitting cigarettes such as peer telephone counselling interventions [21] or internet-based interventions [22], compared to solely raising awareness about the danger of cigarette smoking.

Several limitations must be noted as well. Although this was a two-centered study, we cannot guarantee that our results would be representative of the rest of Croatia. There was a potential problem with selection bias, as patients who refused to answer the questionnaire might have different smoking habits. However, our data seemed to be similar to data found by the Croatian Public Health study in 2015. Another limit of the study is that it did not differentiate between different types of smoking (e.g., e-cigarettes, vaping, tobacco, etc), and did not evaluate the cessation method specifically. The study was prolonged due to technical issues and a simple questionnaire was constructed in order to increase compliance rather than using pre-existing questionnaires such as the World Health Organization one, which might hamper potential comparisons between different populations. Additionally, since the questionnaire collected data that cannot be verified, we depended on the honesty of our patients to obtain exact information. Potentially, having a non-anonymous questionnaire might be responsible for lower accuracy, especially regarding mitigating smoking habits. However, our results still showed dramatically low smoking cessation, regardless of any possible mitigation.

## 5. Conclusions

Although the majority of cancer patients reduced the number of cigarettes smoked, a cancer diagnosis was associated with smoking cessation in only 15.6% of patients. A higher proportion of smoking cessation was recorded in males, younger patients, and patients with smoking-related cancers. However, an alarming rate of continued smoking during cancer treatment was recorded for younger patients and breast cancer patients. Based on our data, we suggest that medical professionals, especially in developing countries, should place a greater spotlight on smoking cessation after a cancer diagnosis. A particular focus should be placed on breast cancer patients and younger patients as both of these groups could yield significant clinical benefits from smoking cessation but are not usually the main focus of anti-smoking campaigns. Further research in developing countries should be undertaken to confirm the issue of high prevalence in the beforementioned groups and evaluate the success of further anti-smoking campaigns.

## Figures and Tables

**Table 1:** General information on the patients who completed the questionnaire (*N* = 695) ^1^.

Patient Characteristic	All Patients	Smoked at Any Period	%
*N*	695	441	63
The highest level of education			
Elementary school	125	79	63
High school	392	258	66
College or higher	109	56	51
Gender			
Male	335	251	75
Female	359	189	53
Age group (based on median age)			
<62	322	232	72
≥62 and higher	364	203	56
Primary cancer			
Lung	127	117	92
Breast	168	82	49
Colorectal	103	60	58
Prostate	48	25	52
Ovarian	36	15	42
Non-ovarian gynecological	32	18	56
Hepatobiliary	25	15	60
Upper gastrointestinal	22	15	68
Head and neck	21	21	100
NET & GIST	19	11	58
Mesothelioma	18	9	50
Brain	16	10	63
Kidney and bladder	15	9	60
Non-specified	14	14	100
Testicular	11	9	82
Melanoma	7	3	43
Sarcoma	6	4	67
Unknown origin	5	3	60
Mediastinal	2	1	50
Metastatic status			
No metastases	377	224	59
Metastatic	304	203	67
Relation of cancer to smoking			
Less smoking related	500	276	55
Smoking-related ^2^	195	165	85

^1^ Some of the data is missing due to patient omissions in the questionnaire. ^2^ Lung, head and neck, cervix, bladder, and kidney.

**Table 2:** Further analysis of the characteristics of the patients who admitted to smoking at any period (*N* = 441).

Patient Characteristic	Smoked at the Time of Canc. Dg.	%	Smokes During Treatment	%	Difference in Proportions (*p*)
Total number	230	52	194	44	
Highest level of education					
Elementary school	44	56	41	52	0.453
High school	139	54	110	43	**<0.001**
College or higher	23	41	18	32	0.125
*Chi-square*	χ^2^ = 3.451 *p* = 0.178		χ^2^ = 5.259 *p* = 0.072		
Gender					
Males	130	52	100	40	**<0.001**
Females	100	53	94	50	0.286
*Chi-square*	χ^2^ = 0.054 *p* = 0.816		**χ^2^ = 4.282 *p* = 0.039**		
Age group (based on median age)					
<62	147	63	123	53	**0.001**
≥62 and higher	80	39	70	34	0.064
*Chi-square*	**χ^2^ = 24.489 *p* < 0.001**		**χ^2^ = 12.09 *p* < 0.001**		
Primary cancer site					
Lung	65	56	50	43	**0.001**
Breast	47	57	45	55	0.754
Colon and rectum	21	35	19	32	0.754
*Chi-square*	**χ^2^ = 8.418 *p* = 0.015**		**χ^2^ = 7.717 *p* = 0.021**		
Metastatic status					
No metastases	101	45	94	42	0.167
Metastatic	117	58	91	45	**<0.001**
*Chi-square*	**χ^2^ = 6.708 *p* = 0.010**		χ^2^ = 0.356 *p* = 0.551		
Relation of cancer to smoking					
Less smoking related	131	47	119	43	**0.045**
Smoking-related	99	60	75	46	**<0.001**
*Chi-square*	**χ^2^ = 6.504 *p* = 0.011**		χ^2^ = 0.356 *p* = 0.551		

The McNemar test was used to describe differences in proportion. Bolded values denote statistical significance (*p* < 0.05). Percentages of patients with a certain characteristic are calculated dividing with ever-smokers. Some of the data is missing due to patient omissions in the questionnaire.

## Data Availability

Full data except patient names is available upon justified request and after Institutional review.

## Appendix A. Includes the Original Questionnaire Translated to English

*The goal of this questionnaire is to evaluate habits regarding smoking, and exploring whether cancer diagnosis changes smoking habits.* *The research will be performed based on modern bioethical standards with respect on respecting your privacy and protecting the secrecy of your medical data.* *Your name and surname is only used to evaluate your initial disease status and WILL NEVER BE PUBLISHED.* *Your privacy is paramount to us, and when we publish the results, all the data WILL BE ANONYMOUS.* *The research was approved by the Ethical Committee of Clinical Hospital Center.*

                     **Anketa je na zaokruživanje!**

NAME AND SURNAME:

YEAR OF BIRTH:

HIGHEST COMPLETED LEVEL OF EDUCATION:   primary school   high school   college or higher

GENDER:   male     female

1. Did you regularly smoke cigarettes **at ANY period** before cancer diagnosis?

**  YES           NO (end of questionnaire)**

2. How long did you smoke in total?

0–5 years     5–10 years     10–20 years     20 years or more

3. Did you smoke cigarettes at the time of your cancer diagnosis?

**  YES (if yes, move to question 4)           NO (if no, move to question 6)**

4. How did cancer diagnosis change your smoking habits?

**I completely quit smoking    I reduced the number of cigarettes     Did not change smoking habits**

(**if yes, move to question 7)**    (**if yes, move to question 5)**        (**if yes, move to question 5)**

5. If you only REDUCED the number of cigarettes or DID NOT CHANGE smoking habits **after** cancer diagnosis, what is the reason that you did not quit completely? (more answers are accepted)
  (A) It is hard to stop
  (B) I do not believe smoking cessation has any effect on cancer treatment
  (C) I do not believe smoking cessation has any effect on prognosis
  (D) Other _____________________________________________________
6. How many cigarettes a day do you smoke NOW?

0     1–5     5–10     10–20     20–30     30 or more

7. How many cigarettes a day did you smoke BEFORE cancer diagnosis?

0     1–5     5–10     10–20     20–30     30 or more
